# Correction: Krauss, J., et al. *Epichloë* Endophyte Infection Rates and Alkaloid Content in Commercially Available Grass Seed Mixtures in Europe. *Microorganisms* 2020, *8*, 498

**DOI:** 10.3390/microorganisms8101616

**Published:** 2020-10-21

**Authors:** Jochen Krauss, Veronika Vikuk, Carolyn A. Young, Markus Krischke, Martin J. Mueller, Katja Baerenfaller

**Affiliations:** 1 Department of Animal Ecology and Tropical Biology, University of Würzburg, 97074 Würzburg, Germany; veronika.vikuk@uni-wuerzburg.de; 2 Noble Research Institute LLC, Ardmore, OK 73401, USA; cayoung@noble.org; 3 Department of Pharmaceutical Biology, Metabolomics Core Unit, University of Würzburg, 97082 Würzburg, Germany; krischke@biozentrum.uni-wuerzburg.de (M.K.); martin.mueller@biozentrum.uni-wuerzburg.de (M.J.M.); 4 Swiss Institute of Allergy and Asthma Research (SIAF), University of Zurich, and Swiss Institute of Bioinformatics (SIB), 7265 Davos, Switzerland; katja.baerenfaller@siaf.uzh.ch

The authors wish to make the following correction to this paper [1]:

After the publication of the manuscript, the authors recognized that there is a discrepancy in Table 1 in five seed mixture compositions (S_10, S_13, S_17, S_29 and S_33), due to differences between the information in catalogs and the actual product labels. The authors changed the seed compositions and suppliers, where necessary, to match with product labels on the seed mixture packages. We also deleted two sentences from the discussion regarding speculations on possible infections of the seed variety NEW ORLEANS, which was not a component in any of the tested seed mixtures.

We changed Table 1 and present the correct varieties here.

These inconsistencies occurred as the information on seed varieties were wrongly taken from catalogs instead of package labels. We want to point out that contaminations with *Epichloë* infected seeds could have occurred at different production steps of the seed mixtures (breeders, producer of seed cultivars, producer of the seed mixture, trader, shop/market, etc.). Therefore, our study cannot be used to detect the source of the *Epichloë* contaminations of the seed varieties.

The authors want to point out that seed mixture S_33 did not contain the perennial ryegrass variety NEW ORLEANS, which was therefore not tested. Speculations on possible infections of the variety NEW ORLEANS in the discussion are not valid and we apologize for this unjustified speculation.

Therefore the authors would like to delete these sentences from the discussion: “The turfgrass seed mixture S_33 contains 5% of the perennial ryegrass variety NEW ORLEANS, which is listed as a top American breeding variety and could be the source of *Epichloë* infected seeds. The low percentage of this variety in the seed mixture could result in sampling variation, which may explain why 8.7% of E+ seeds were detected, and why the alkaloid quantification results differed between the two laboratories.”

Following a reviewer’s comment, we meanwhile grew plants from the four grass seed mixtures (S_10, S_24, S_32 and S_33) which were *Epichloë* infected and contained alkaloids, and tested 8- and 20-week old seeded plants for *Epichloë* alkaloids. We used 30 randomly picked seeds per seed mixture and tested eight grass samples containing about ten randomly picked grass tillers per seed mixture. In S_10 and S_33 we detected peramine and lolitrem B. In S_32, we detected peramine, lolitrem B and in addition ergovaline. In S_24, we could not detect alkaloids in the plants. As we only picked 30 random seeds, it could be possible that no infected seeds were grown from S_24. As three of the four endophyte and alkaloid positive seed mixtures produced alkaloids in the grown plants, we confirm our result that infected seed mixtures can introduce vertebrate toxic alkaloids into the agricultural environment.

The authors would like to apologize for any inconvenience caused to the readers by these changes.

## Figures and Tables

**Table 1:** Composition of grassland seed mixtures, bold indicate seed mixtures with infections of *Epichloë* spp. and detected vertebrate toxic alkaloids. Letters in brackets after the product name indicate if seed mixtures are mainly used as forage grass (F) or turf grass (T) according to supplier information. Supplier names are in italic. **Changed information is highlighted in grey.**

ID	Perennial Ryegrass Varieties	Tall Fescue Varieties	Other Grasses	Product
S_10 *	**30 % BARCLAY II** **15 % BAREURO** **20% BARLICUM** **20% BARLIBRO** **15 % BAROMARIO**	-	-	**Regenerations-Mischung RPR, *Eurogreen,Barenbrug*** **(T)**
S_11	Unknown variety in unknown percentage	-	*Poa pratensis, Festuca pratensis,* *Dactylis glomerata, Phleum pratense, Festuca rubra, Agrostis*	Gräsermischung Weidesaat, *Kräuterwiese* (F)
S_12	25% lawn type, unknown variety 25% pasture type, unknown variety	-	20 % *Poa pratensis*, 20 % *Phleum pratense*, 10 % *Festuca rubra*	Country Horse 2117, *DSV* (F)
S_13 *	14 % CARNAC 21 % EURODIAMOND 7 % DOUBLE 4n 8 % FABIAN 4n 8 % CSI CORSICA 17 % ZÜRICH	-	25 % *Poa pratensis*	Regeneration Highspeed, *UFA* (T)
S_14	-	100 % LIPALMA	-	*Camena Samen* (F)
S_15	33 % MATHILDE 34 % WADI 33 % BELIDA	-	-	Elite Gvo (ELITE Grünland Nr. 5), *Rudloff* (F)
S_16 ^#^	20 % EURODIAMOND, 15 % SIRTAKY	15 % BARCESAR, 35 % MEANDRE	15 % *Poa pratensis*	Reitbahn, *UFA* (T)
S_17 *	25 % ASTONHOCKEY	20 % HYKOR	25 % *Festuca pratensis*, 20 % *Phleum pratense*, 10 % *Poa pratensis*	Country Öko 2217, *DSV* (F)
S_18	15 % BOYNE 20 % TODDINGTON 20 % INDICUS 1 15 % POLIM 15 % ARUSI 15 % GARBOR	-	-	Profi Nachsaat Gvo, *Tystofte Fonden* (F)
S_19	100 % KARATOS	-	-	*Camena Samen* (F)
S_20	-	-	7 % *Agrostis capillaris*, 3 % *Alopecurus pratensis*, 12 % *Arrhenatherum elatius*, 10 % *Cynosurus cristatus*, 10 % *Dactylis glomerata*, 15 % *Festuca rubra*, 1 % *Holcus lanatus*, 1 3 % *Phleum pratense*, 18 % *Poa pratensis*, 1 % *Trisetum flavescens*	Heuwiese für Pferde, *Appels Wilde Samen* (F)
S_21	10 % KARATOS 20 % KUBUS, 15 % TWYMAX	-	25 % *Phleum pratense* 12 % *Poa pratensis* 15 % *Festuca rubra*	Pferdeweide 1, *Camena Samen* (F)
S_22	8% PREMIUM	-	18% *Festulolium*, 18% *Phleum pratense*, 15% *Festuca pratensis*	Rotkleegras 91, *Camena Samen* (F)
S_23	100 % POLIM	-	-	*Camena Samen* (F)
**S_24**	**12 % BELLEVUE** **20 % BOYNE** **40 % STEFANI**	-	**18 % *Phleum pratense*** **10 % *Poa pratensis***	**Pferdeweide Nachsaat, *Raiffeisen* (F)**
S_25	20 % IVANA 10 % TIVOLI 20 % SW BIRGER	20 % SWAJ	10 % *Poa pratensis* 20 % *Phleum pratense*	Pferdegreen Öko PR940, *BSV Saaten* (F)
S_26	100 % TWYMAX	-	-	*Camena Samen* (F)
S_27	28 % MATHILDE, 23 % ALFAN, 13 % BELIDA	-	10 % *Festuca pratensis*, 5 % *Poa pratensis*, 21 % *Phleum pratense*	Elite 20, *Rudloff* (F)
S_28	25 % MARAVA 30 % BOKSER 30 % WADI	-	15 % *Phleum pratense*	Equitana Nachsaat Gvo, *Rudloff* (F)
S_29 *	10 % TURFGOLD	45 % BARCESAR, 25 % DEBUSSY 1	20 % *Poa pratensis*	Monaco-Mischung RSM, *Eurogreen* (T)
S_30	11 % TREND 5 % KARATOS 10 % TWYMAX	-	10 % *Festuca pratensis*, 11 % *Festulolium fedoro*, 7 % *Dactylis glomerata*, 5 % *Poa pratensis*, 5 % *Festuca rubra*, 14 % *Phleum pratense*	Kräuterweide, *Camena Samen* (F)
S_31	Unknown variety in unknown percentage	-	*Festuca pratensis, Poa pratensis, Poa trivialis, Festuca rubra, Phleum pratense, Alopecurus pratensis, Cynosurus cristatus, Elymus repens*	Pferdeweide-Reparatursaat, *Kräuterwiese* (F)
**S_32**	**15 % MARAVA** **15 % BOKSER** **15 % WADI**	-	**25 % *Phleum pratense*** **20 % *Poa pratensis*** **10 % *Festuca rubra***	**Equitana Universal, *Rudloff* (F)**
S_33 *	**9 % COLUMBINE** **7 % DOUBLE/FABIAN** **12% ZURICH** **5 % CSI CORSICA** **12 % SIRTAKY**	-	**40 % *Poa pratensis*,** **15 % *Festuca rubra***	**Primera Highspeed, *UFA* (T)**

* Seed composition as indicated on package different to that in online catalogs. # Seed composition not indicated on package.
