# Supplementary material for: Epichloë Endophyte Infection Rates and Alkaloid Content in Commercially Available Grass Seed Mixtures in Europe
Source: Microorganisms. 2020 Mar 31;8(4):498. doi: 10.3390/microorganisms8040498 (PMC7232243; doi:10.3390/microorganisms8040498)

Figure S1: A composite image representing examples of endophyte infected samples, S_30 and S_33, as tested by a multiplex PCR reaction. S_30: banding pattern characteristic for *Epichloë uncinata* in *Festuca pratensis*, S_33: banding pattern characteristic for *Epichloë festucae* var. *lolii* in *Lolium perenne*. PCR marker on the right is the 1 kb+ ladder (Thermo Fisher Scientific). On the left the PCR marker fragments for each gene are indicated, *tefA* = a conserved gene encoding the translation elongation factor 1-alpha; *perA-T2* = a marker to the *perA* gene encoding peramine synthetase second thiolation domain, *lolC* = a loline gene marker, *dmaW* = an ergot alkaloid gene marker, *idtG* = indole-diterpene gene marker.

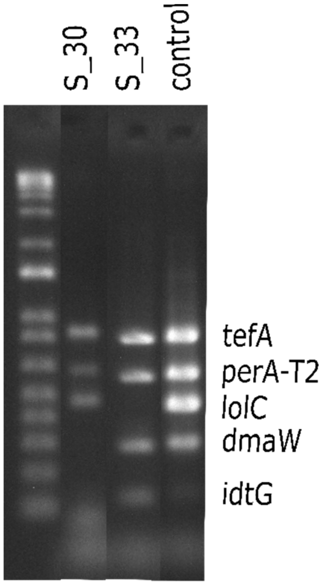

Supplement: Supplementary file 1 [file microorganisms-08-00498-s001.pdf]
